# Odd Sensation Induced by Moving-Phantom which Triggers Subconscious Motor Program

**DOI:** 10.1371/journal.pone.0005782

**Published:** 2009-06-03

**Authors:** Takao Fukui, Toshitaka Kimura, Koji Kadota, Shinsuke Shimojo, Hiroaki Gomi

**Affiliations:** 1 NTT Communication Science Laboratories, Nippon Telegraph and Telephone Corporation, Morinosato, Atsugi, Kanagawa, Japan; 2 ERATO Shimojo Implicit Brain Function Project, Japan Science and Technology Agency, Atsugi, Kanagawa, Japan; 3 Division of Biology, California Institute of Technology, Pasadena, California, United States of America; Victoria University of Wellington, New Zealand

## Abstract

Our motor actions are sometimes not properly performed despite our having complete understanding of the environmental situation with a suitable action intention. In most cases, insufficient skill for motor control can explain the improper performance. A notable exception is the action of stepping onto a stopped escalator, which causes clumsy movements accompanied by an odd sensation. Previous studies have examined short-term sensorimotor adaptations to treadmills and moving sleds, but the relationship between the odd sensation and behavioral properties in a real stopped-escalator situation has never been examined. Understanding this unique action-perception linkage would help us to assess the brain function connecting automatic motor controls and the conscious awareness of action.

Here we directly pose a question: Does the odd sensation emerge because of the unfamiliar motor behavior itself toward the irregular step-height of a stopped escalator or as a consequence of an automatic habitual motor program cued by the *escalator itself*. We compared the properties of motor behavior toward a stopped escalator (SE) with those toward moving escalator and toward a wooden stairs (WS) that mimicked the stopped escalator, and analyzed the subjective feeling of the odd sensation in the SE and WS conditions. The results show that moving escalator-specific motor actions emerged after participants had stepped onto the stopped escalator despite their full awareness that it was stopped, as if the motor behavior was guided by a “phantom” of a moving escalator. Additionally, statistical analysis reveals that postural forward sway that occurred after the stepping action is directly linked with the odd sensation.

The results suggest a dissociation between conscious awareness and subconscious motor control: the former makes us perfectly aware of the current environmental situation, but the latter automatically emerges as a result of highly habituated visual input no matter how unsuitable the motor control is. This dissociation appears to yield an attribution conflict, resulting in the odd sensation.

## Introduction

**Figure 1:**
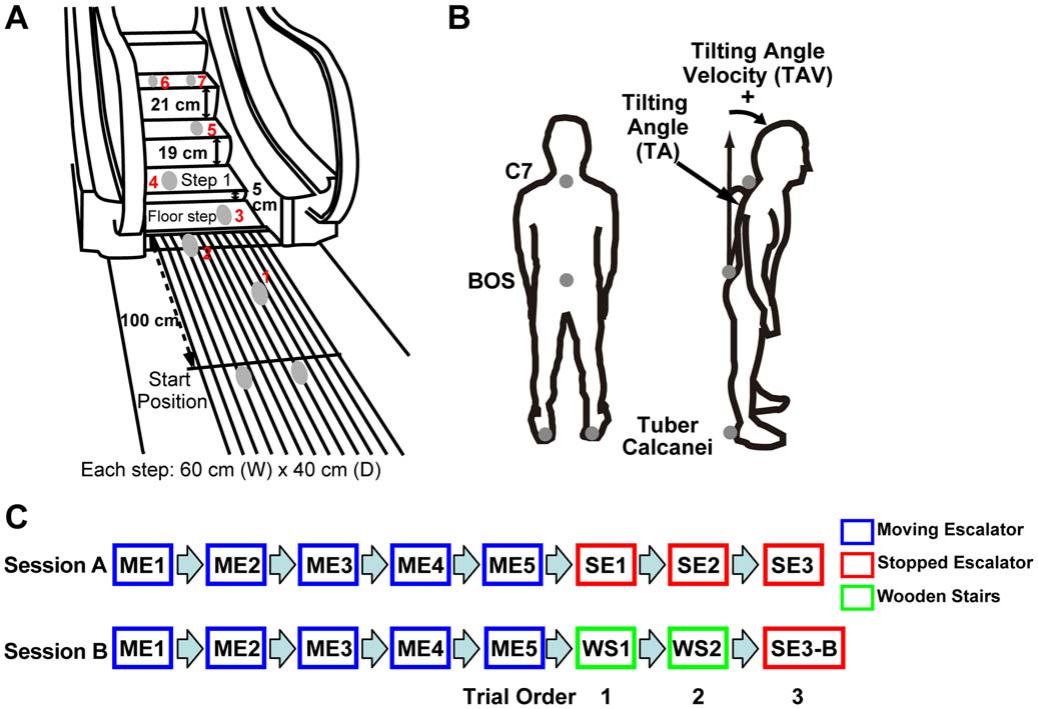
Experimental setup. (A) Configuration of stopped escalator and gait pattern shown by gray ellipses (the case of gait initiation with right leg). The number beside the ellipse indicates the order of gait steps in one trial. The physical dimensions of four steps (i.e. the shorter first step) and approach was duplicated by wooden stairs and plastic material. The stairs have no hand rail. (B) Back and lateral views of participants. Markers were placed C7 of the spine, basis ossis sacri (BOS), and right and left heels (gray points). As indices of postural sway, we used tilting angle (TA) defined as the angle made by the line C7 connecting with the BOS and vertical axis and tilting angle velocity (TAV) obtained by differentiating the TA. (C) The experimental sessions. In session *A*, five consecutive moving escalator trials (ME1–ME5) were followed by three consecutive trials of stepping onto stopped escalator (SE1–SE3) as one block. In session *B*, participants performed five moving escalator trials, two wooden stairs trials (WS1, WS2), and finally a stopped escalator trial (SE3-B) sequentially as one block. A total of 16 blocks were done for each session.

We usually perform actions in daily life appropriately according to the situations we encounter. However, despite our being in a situation where we adequately understand the external environment and have a specific action intention and the motor skills to perform the action, we sometimes feel our movements are not properly performed. A familiar example for urban people is the action of stepping onto a stopped escalator. When we encounter an escalator that is out of service, we recognize the escalator is certainly stopped and is not going to move; have the definite intention to step onto the stopped escalator (action to peculiar physical dimensions of stairs, see Fig. 1A), not a moving one (very familiar action); and then step onto it. After doing so, despite the proper awareness and intention, we frequently feel clumsy, as if our posture is awkward, our bodies heavier, and the steps are sometimes hard to climb. This clumsiness is associated with some kind of odd sensation.

This sensation would be different from the feeling one gets from a simple action error. For example, when going up or down stairs, we sometimes extend the foot in expectation of an additional step at the end of the stairs, which causes us to stagger. In such a case, we also feel we did not properly perform, but we are able to easily attribute losing our footing to a self-prediction error caused by our thinking there was an additional step. On the contrary, when we step onto a stopped escalator even with perfect knowledge that it is stationary, we are often surprised by the strange feeling accompanied by our own action. This feeling implies the involvement of subconscious processes *against* conscious awareness in the emergence of the odd sensation. Thus, this phenomenon raises an intriguing question as to how implicit motor programming escapes from conscious top-down control and offers an opportunity to study it.

This unique phenomenon experienced in a stopped-escalator situation has been a subject of general interest, but it has only been investigated in its visual aspects, such as the potential effect of periodical surface grooves in steps on depth perception [1], [2], and its motor ones (short-term adaptation in sled walking e.g., [3]). In a series of studies by Bronstein and colleagues [3]–[6], participants in a locomotor adaptation task using a mobile sled reported a sensation similar to the one associated with a stopped escalator, but this sensation was just descriptive. That is, it remains unclear why the odd sensation is induced when stepping onto a stopped escalator. In the present study, we used a real escalator to investigate the occurrence of the odd sensation qualitatively to identify specifically the relationship between behavioral properties and the perception of the odd sensation.

There are three possible explanations for the emergence of the odd sensation when stepping onto a stopped escalator.

1. The odd sensation concurrently but independently occurs with the postural or leg behavioral properties. That is, the sensation has nothing to do with the body sensation derived from such behavioral properties. For instance, simple unfamiliarity with encountering a stopped escalator could induce the sensation.
2. The odd sensation occurs due to the unique height of the steps, in which the first step is shorter than others. This unusual step nonuniformity induces clumsiness because we do not get used to such nonuniformity, and the clumsiness leads to the odd sensation.
3. The odd sensation results from an inappropriate action inconsistent with the current situation despite the proper understanding of the situation. Stepping onto a moving escalator is a highly habituated action, so the habitual motor program for a *moving* one would emerge even when we step onto a *stopped* one. The subconscious emergence of the habitual escalator-specific motor program leads to the inappropriate motor behavior, which leads to the odd sensation.

All of the above possibilities focus on the relationship between the odd sensation and body sensation derived from the motor behaviors (i.e. stepping onto and climbing a stopped escalator). The odd sensation could reflect the dissociation between the subconscious motor control and conscious awareness, so investigating this relationship will deepen our understanding of interactions between automatic/subconscious and conscious processes towards action.

To determine which possibility is more likely, we compared the kinematic properties of the lower limbs and upper body (Fig. 1B) when participants stepped onto a stopped escalator compared with those when they stepped onto a moving escalator and onto a wooden stairs duplicating the physical dimensions of a stopped escalator (Fig. 1A). Any difference in the results would indicate whether the motor behaviors in a stopped escalator are triggered by the nonuniformity of the step-height or by the habitual action program. We also analyzed the subjective feeling of the odd sensation reported after trials on the stopped escalator and wooden stairs with a comparable step-height (i.e., smaller height of the first step). The second explanation above predicts the emergence of the odd sensation even when stepping onto the wooden stairs. The first and third ones predict that the odd sensation only occurs for a stopped escalator, and the third one further predicts the emergence of the habitual motor program as a necessary condition for the odd sensation. We further explored the causal relation by statistical path analysis using structural equation modeling [7].

Our results show that i) the odd sensation emerges only in a stopped-escalator situation, ii) the habitual escalator-specific motor program emerges *not before but after* stepping onto a stopped escalator, and iii) the habitual motor program triggers drastic forward postural sway, which is closely related to the odd sensation score, suggesting that the odd sensation is tightly linked to the automatic motor actions that implicitly emerge.

## Results

First, we examined whether the stopped-escalator step-height (the shorter first step) contributes to the emergence of the odd sensation. If it does, the odd sensation would be felt even on a wooden stairs (WS) with physical dimensions identical to those of a stopped escalator. We also verified how the perception of this sensation varies with the repetition of stopped-escalator (SE) trials and with the insertion of a different situation (i.e., wooden stairs). Second, we analyzed the behavioral properties for the stopped escalator by comparing them to those for the moving escalator (ME) and wooden stairs, focusing on whether specific motor behaviors that we empirically know (e.g., stumbling) appeares. Finally, we examined the relationship between motor behaviors and the odd sensation.

We conducted two experimental sessions; each session consisted of 16 blocks of eight successive trials as follows (Fig. 1C). In session *A*, five consecutive ME trials (ME1–ME5) were followed by three consecutive SE trials (SE1–SE3). In session *B*, participants performed five ME trials, two WS trials (WS1, WS2), and finally a SE trial (SE3-B) sequentially. For data analysis, each trial was grouped by the trial order in each session, and the grouped trials (i.e., 16 trials) in each condition determined by the session and trial order were named as follows: SE1, SE2, SE3 conditions in session *A*; WS1, WS2, SE3-B conditions in session *B*. ME5 condition in session *A* was analyzed as the typical condition of habitual moving escalator situation (see **Material and Methods** for details).

### Odd sensation for the stopped escalator did not emerge for wooden stairs

**Figure 2:**
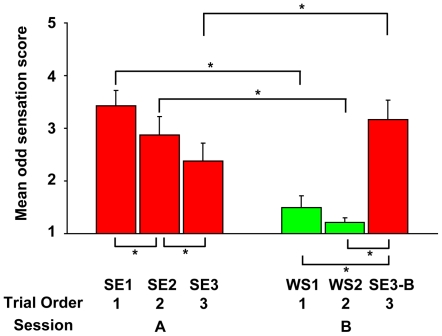
Perception of odd sensation. Significant differences between SE1 and WS1 conditions and between SE2 and WS2 conditions suggest participants surely felt the odd sensation when they stepped onto a stopped escalator. Mean scores in WS1 and WS2 conditions were nearly 1, suggesting the structural step nonuniformity (the shorter first one) was not essential for the perception of the odd sensation. Mean score in the SE3 condition was significantly lower than in SE1, suggesting that the more we experience stepping onto a stopped escalator, the less strongly we feel the odd sensation. There was also a significant difference between SE3 and SE3-B conditions. These observations suggest that the visual context (escalator vs. wooden stairs) in which the action is taken, rather than the irregular step-height itself, is essential for the perception of the odd sensation. Asterisks represent p<0.05.

As shown in Fig. 2, participants reported lower scores (1 or 2, which indicate no or almost no odd sensation) in most trials for WS1 and WS2 conditions [mean scores: 1.50 (SE = 0.22), 1.21 (SE = 0.10) respectively; no significant differences between them], indicating that irregular step-height itself does not cause the odd sensation. A significant interaction between session (A and B) and trial order (1–3) was observed (two-way repeated measures ANOVA, F(2, 12) = 60.52, p<.001) and the mean odd sensation score in the SE3-B condition was significantly higher than in the SE3 condition. These results suggest that the adaptation to the step-height itself did not completely eliminate the odd sensation, indicating the visual context (escalator vs. wooden stairs) in which the action is taken is critical for the emergence of the odd sensation.

The gradual decrease of the odd sensation score from the SE1 to SE3 conditions suggests that the more we experience stepping onto a stopped escalator, the less we feel the odd sensation. This result is in line with our intuition and may reflect adaptation to the situation in which we step onto a stopped escalator.

### Motor behavior altered *after* stepping onto the stopped escalator

**Figure 3:**
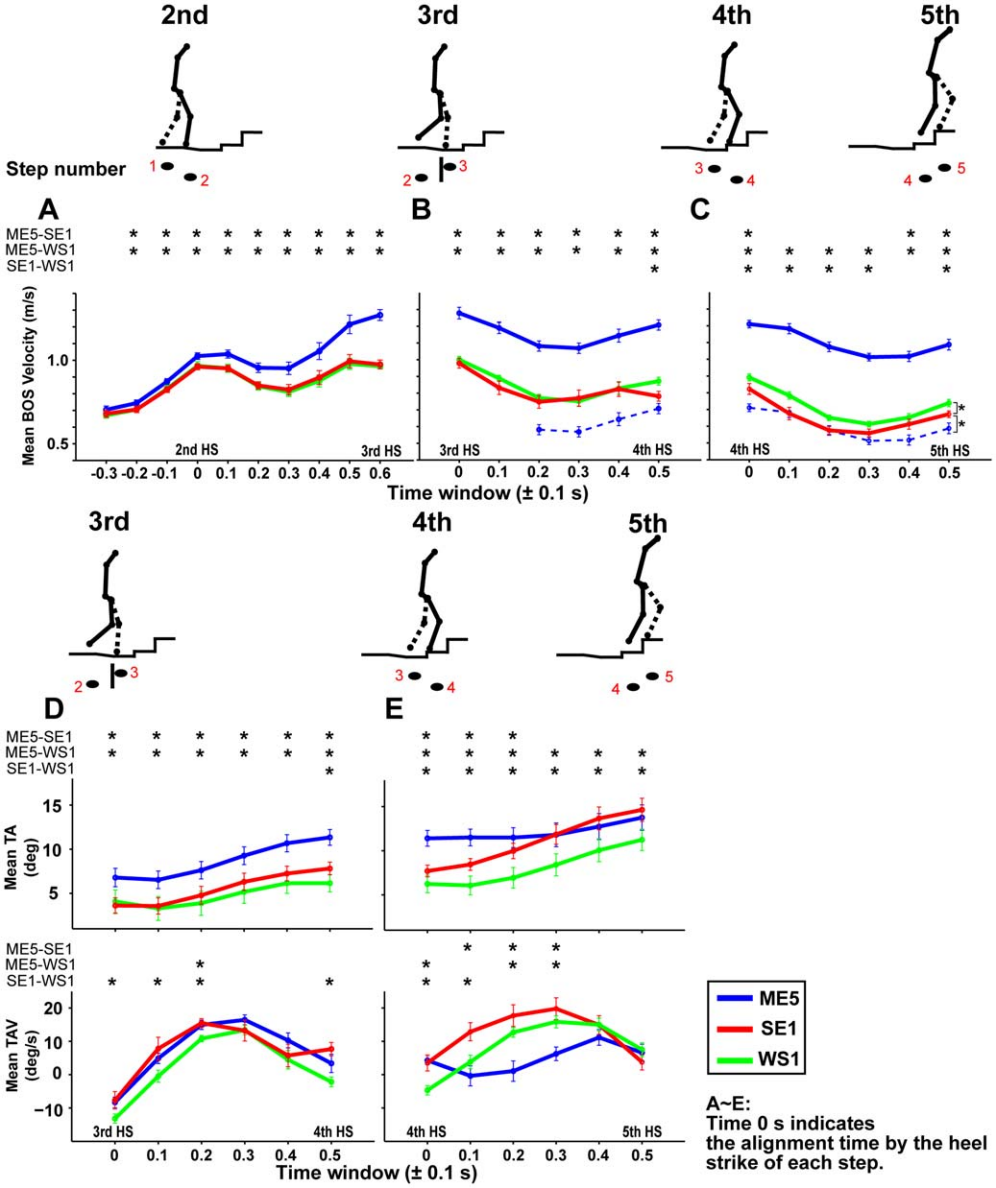
Temporal variations of walking velocity (BOS), TA and TAV aligned by the time of each heel strike. Mean 0.2 s time window horizontal velocity of basis ossis sacri (BOS) (as walking velocity) in three conditions (blue, red, green lines indicate ME5, SE1, WS1 conditions, respectively) were aligned by the heel strike of the (A) second, (B) third, and (C) fourth steps. Time 0 indicates the time of the heel strike of each step, each time (abscissa axis) ±0.1 s represents a 0.2 s time window. Note that the 0.6 in Fig. 3A and the 0 in Fig. 3B and the 0.5 in Fig. 3B and the 0 in Fig. 3C were temporally overlapped. Blue dotted lines in Figs. 3B and C indicate the actual BOS velocities in the ME5 condition after stepping which were calculated by subtracting escalator speed itself (0.5 m/s) from measured BOS velocity. Tilting angle (TA) and tilting angle velocity (TAV) temporally averaged in each 0.2 s time window, aligned by the heel strike of the (D) third and (E) fourth steps. As in Fig. 3B and C, time 0 indicates the time of the heel strike of each step, and 0.5 in Fig. 3D and 0 in Fig. 3E were temporally-overlapped. Action sequences are shown by stick figures above each figure. 2nd HS, 3rd HS, 4th HS, and 5th HS in Figs. 3A–3E indicate the approximate time of the heel strikes of the second, third, fourth, and fifth steps in each figure.

When we step onto a moving escalator, an increase of walking velocity before the stepping action would be required for additional propulsion for the stabilization of posture after the action. With the increase of walking velocity, postural forward tilting occurs to maintain the balance of the whole body. We therefore first examined whether the walking velocity before the stepping action in the moving-escalator situation significantly increases compared to that in the wooden-stairs situation and whether postural forward tilting increases with walking velocity. Next, we examined whether walking velocity (Fig. 3A) and postural forward tilting (Fig. 3D) in the stopped-escalator situation (red line in each figure) as well as that in the moving-escalator situation (blue line) significantly increase compared to that in the wooden-stairs situation (green line).

Participants stepped onto an escalator or the wooden stairs with their third step. The velocity of basis ossis sacri in the horizontal direction (BOS velocity; see Fig. 1B) was measured as the walking velocity, and the tilting angle (TA) and tilting angle velocity (TAV) were calculated as the indices of postural forward tilting (see Fig. 1B). Mean values of walking velocity, TA, and TAV in 0.2 s time windows aligned by the heel strike of each step were calculated (Figs. 3A–3E). Hereafter, the data at time T represents the temporal mean value for the time window of ±0.1 second. As shown in Fig. 3A, the velocity in the SE1 condition (indicated by a red line) before stepping onto the escalator, which is of primary interest in this experiment, showed no significant differences from that in the WS1 condition (green) in all phases from −0.3 to 0.6 in Fig. 3A and was significantly lower than that in the ME5 condition (blue) from the −0.2 in Fig. 3A. That is, the SE1 velocity pattern (red) before stepping is remarkably similar to that in the WS1 condition (green). As for postural forward tilting (Fig. 3D), the mean TA, like the walking velocity, in the SE1 condition (red) is no different from that in the WS1 condition (green) and is already significant from those of the ME5 condition (blue) at 0. These results suggested that, before we step onto a stopped escalator, our visuomotor system performs appropriately in accordance with the external environment (i.e., with the fact that the escalator is stopped).

However, such appropriate motor behavior, which was consistent with the current external environment, changed after the stepping action. Walking velocity in the SE1 condition (red in Fig. 3C) significantly decreased compared to that in the WS1 condition (green) just after the fourth step (at 0 in Fig. 3C) and approached that in the ME5 condition (blue dotted line calculated by subtracting escalator speed itself (0.5 m/s) from measured walking velocity at 0.1, 0.2 in Fig. 3C). In addition, as shown in Fig. 3D, postural forward sway (TAV) in the SE1 condition (red) increased more than it did in the WS1 condition (green) after stepping (TAVs at 0.1 and 0.2 in Fig. 3D), and the TAs in the SE1 condition (red) were significantly greater than those in the WS1 condition (green) from 0.5 in Fig. 3D. The increase of TAV in the SE1 condition was remarkable after the heel strike of the fourth step. Specifically, TAVs in SE1 condition (red) at 0 and 0.1 in Fig. 3E are significantly larger than those in the WS1 condition (green). In summary, a drastic postural forward sway occurred with decreasing of walking velocity in the SE1 condition, but not in the WS1 condition, regardless of the identical step-height (the smaller height of the first step). Therefore, this postural forward sway was not due to the structural nonuniformity of steps but to stepping onto the *escalator* itself, which triggered the escalator-specific motor program, resulting in TAs in the SE1 condition (red) at 0.4 and 0.5 in Fig. 3E comparable to those in the ME5 condition (blue).

Since the steps continuously move in the normal moving-escalator condition, leg movements could be also affected by the behavioral context as well. Therefore, we next focus on whether there were any behavioral differences between the SE1 and WS1 conditions or not.

**Figure 4:**
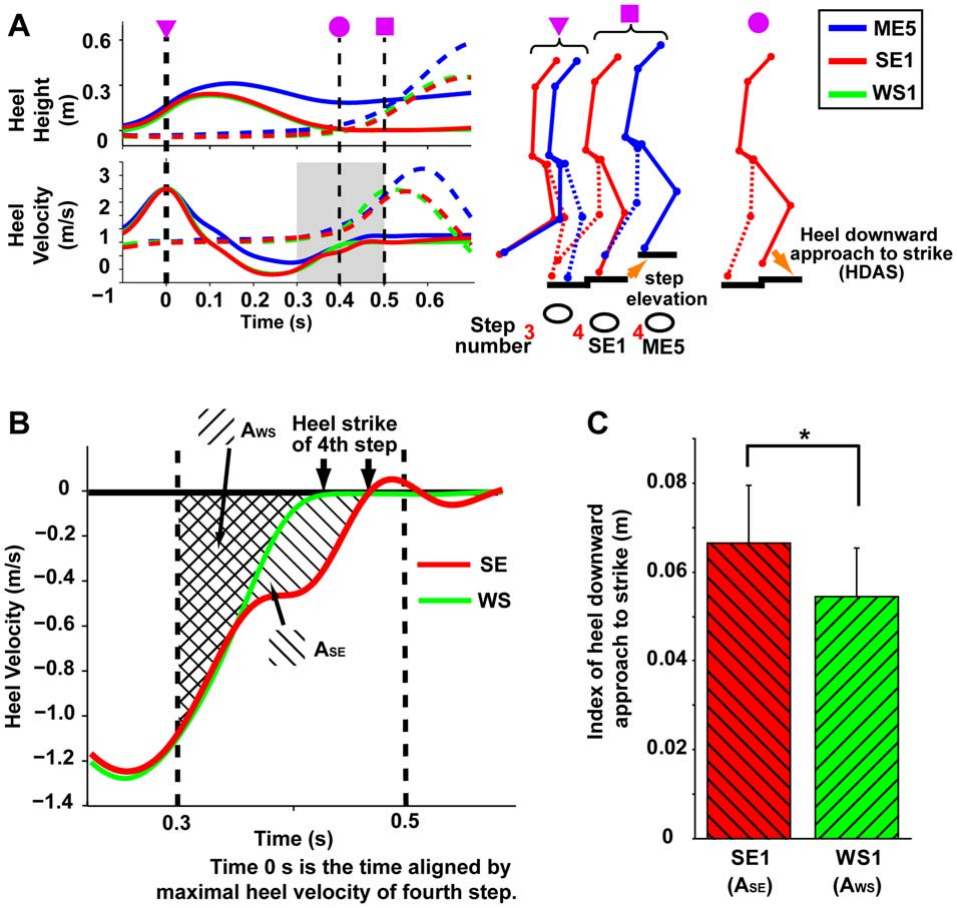
Behavioral properties of lower limbs in moving-escalator, stopped-escalator and wooden-stairs conditions. (A) The representative temporal profiles of heel height, heel vertical velocity aligned by the maximal heel velocity of the fourth step. The magenta symbols (inverted triangle, circle, and square) above the stick figure indicate the time point shown by the same symbol in the temporal profile figure. Each stick figure shows the action sequences to the step elevation in the ME5 condition with those without step elevation in SE1 condition (left) and to the heel's downward approach to the strike (right). (B) Schematic profile of heel vertical velocity in stopped escalator (SE) and wooden stairs (WS) before the heel strike of the fourth step. The time window [0.3–0.5] corresponds to the gray area in Fig. 4A. The temporal profile in stopped-escalator situation showed double decelerations, which may reflect corrective movement before the heel strike (the area A_SE_ indicated by the hatched lines diagonally right downward), while that in wooden-stairs condition showed a single deceleration (the area A_WS_ indicated by the hatched lines diagonally right upward). On the basis of the data aligned by the maximal heel velocity of the fourth step (see black dotted vertical line in Fig. 4A), we calculated area A_SE_ as heel's downward approach to strike (HDAS) for the stopped escalator and area A_WS_ as that for the wooden stairs. (C) The HDAS index in the SE1 and WS1 conditions (see also the stick figure in Fig. 4A). The HDAS in the SE1 condition showed significantly larger than that in the WS1 condition. Asterisks represent p<0.05.

Fig. 4A shows typical temporal profiles of heel height (upper panel) and heel vertical velocity (lower panel) aligned by the maximal heel velocity of the fourth step along with stick figures illustrating the action sequences after stepping. The motor behavior in the SE1 condition was apparently similar to that in the WS1 condition but was actually different in phase immediately before the heel strike (at 0.4 in Fig. 4A). Namely, the heel's downward approach to the strike (HDAS) of the fourth step (see the right stick figure in Fig. 4A) was modified online specifically in the SE1 condition (red curve indicating double downward decelerations before the heel strike in Fig. 4B), despite the identical step height. As the index, we adopted heel's downward approach to the strike using the time-integral of heel vertical velocity, and found that the index in the SE1 condition (A_SE_ in Fig. 4B) was significantly greater than that in the WS1 condition (A_ws_ in Fig. 4B) as compared in Fig. 4C [F(1, 6) = 7.75, p<.05]. This suggests that, in the stopped-escalator situation, a habitual escalator-specific motor program anticipating the step elevation emerged regardless of the full awareness that the escalator was stopped. The actual downward movement of the heel was therefore too short to arrive at the step, so corrective lower limb movement was required.

In summary, motor behaviors before stepping were properly adjusted to the external world, reflecting conscious awareness that the escalator was stopped (from visual cues). Yet, contrary to this conscious awareness, the habitual motor program (i.e. the program for moving one) subconsciously emerged after stepping very similarly whether the escalator was moving or stopped. As a result, the actual movements were inconsistent with the current environment when the escalator was stopped, resulting in inappropriate motor responses (postural forward sway and heel's downward approach to the strike).

### The upper body movement mainly contributes to the perception of the odd sensation

As described above, we found that the perception of the odd sensation only occurred in the stopped-escalator situation and that, only after participants had stepped onto the stopped escalator did specific motor behaviors of both lower limb and upper body emerge. Such averaged phenomena, however, do not guarantee the causal relationship between the emergence of the odd sensation and such behavioral properties. To investigate this relationship, we developed a structural equation model, which uses a statistical multivariate technique [7] (see **Material and Methods** for details).

**Figure 5:**
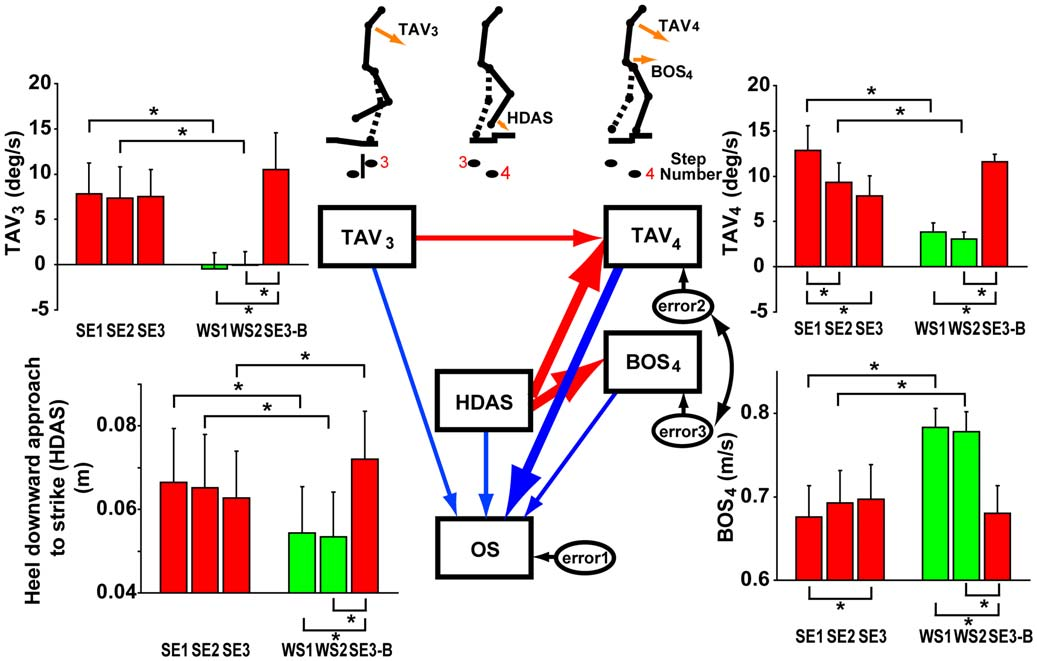
The causal path model and behavioral changes in different conditions. The adopted behavioral properties are as follows: Mean tilting angle velocity (TAV) at 0.1 aligned by the heel strike of the third step (TAV_3_), The heel's downward approach to strike (HDAS), mean TAV at 0.1 aligned by the heel strike of the fourth step (TAV_4_), mean horizontal velocity of the basis ossis sacri (BOS) at 0.1 aligned by the heel strike of the fourth step (BOS_4_). Stick figures show the action sequence and the adopted motor actions schematically. Bar graphs show the mean values of each behavioral index in each condition. Blue lines indicate paths from the behavioral index to the odd sensation score, and red lines indicate paths between behavioral indices, and the width of the path indicates the strength of relationship (the number of participants who showed significant path coefficient, see also Tables 1 and 2). OS stands for the odd sensation score, and error 1–4 is the error term. The inappropriate lower limb movements (HDAS) did not *directly* induce the perception of the odd sensation (except for one participant) but upper body movements (mainly TAV_4_) induced it, although there is a kinematics chain between lower limb and upper body movements (from HDAS to BOS_4_, and from HDAS to TAV_4_).

The center panel of Fig. 5 illustrates the model we used. Here, we adopted four variables as representatives of behavioral changes: heel's downward approach to strike (HDAS), tilting angle velocity at 0.1 aligned by the heel strike of the third step (TAV at 0.1 in Fig. 3D; TAV_3_), tilting angle velocity at 0.1 aligned by the heel strike of the fourth step (TAV at 0.1 in Fig. 3E; TAV_4_), and BOS velocity at 0.1 aligned by the heel strike of the fourth step (BOS at 0.1 in Fig. 3C; BOS_4_). All these behavioral indices showed significant differences between SE1 (SE2) and WS1 (WS2) conditions as shown in bar graphs of Fig. 5. For model simplicity, we excluded the tilting angle at around the heel strike of the fifth step, because this tilting angle could be regarded as an event subsequent to BOS_4_ and TAV_4_. In the center panel, the paths from each behavioral event to odd sensation score (OS) enable us to identify which kinematics feature is essentially correlated with perception of the odd sensation. This path design would reveal whether escalator-specific motor behaviors cause the odd sensation or not. The paths between each kinematic variable were also designed to examine the kinematic chain, especially that between lower limb and upper body movements. Total fitting scores (goodness of the model fitting) were GFI = .979, CFI = .979, RMSEA = .039.

From this analysis, we found a significant causal contribution of the upper body movements to the odd sensation. Table 1 summarizes the standardized path coefficients from each kinematic variable to the odd sensation in the model of Fig. 5 for each participant. Five participants (participants 1, 2, 3, 4, 5) out of 7 showed significant and the highest path coefficients from TAV_4_, although participant 5 also showed a significant path coefficient from HDAS. Participant 7 showed a significant path coefficient from TAV_4_, but his coefficient from TAV_3_ was highest. Participant 6 showed a significant path coefficient from BOS_4_, but the positive influence implies that the more the BOS velocity increased the more he felt the odd sensation. Additionally, as shown in Table 2, we found a kinematic chain between lower limb and upper body movements. Six participants (participants 2, 3, 4, 5, 6, and 7) showed significant path coefficients from HDAS to TAV_4_ and from HDAS and BOS_4_.

**Table 1:** Standardized path coefficients in the SE condition from each kinematics variable to the odd sensation in the model in Fig. 5.

Participant	Path			
	TAV_3_→OS	HDAS→OS	BOS_4_→OS	TAV_4_→OS
1	0.074	0.246+	−0.006	0.443**
2	−0.068	0.160	−0.018	0.331*
3	0.119	−0.054	−0.155	0.339*
4	0.046	−0.055	−0.270	0.367*
5	0.124	0.330*	0.074	0.498**
6	−0.089	0.075	0.363*	0.055
7	0.429**	0.142	0.023	0.352*

TAV_3_: mean tilting angle velocity (TAV) at 0.1 aligned by the heel strike of the third step.

HDAS: heel's downward approach to strike.

TAV_4_: mean TAV at 0.1 aligned by the heel strike of the fourth step.

BOS_4_: mean BOS velocity at 0.1 aligned by the heel strike of the fourth step.

OS: the score of odd sensation.

** p<.01.

* p<.05.

+ p<.1.

**Table 2:** Standardized path coefficients in the SE condition between each kinematics variable in the model in Fig. 5.

Participant	Path		
	TAV_3_→TAV_4_	HDAS→TAV_4_	HDAS→BOS_4_
1	0.087	−0.098	−0.101
2	−0.151	0.275*	−0.543**
3	−0.141+	0.506**	−0.605**
4	0.135	0.353**	−0.509**
5	−0.321**	0.414**	−0.691**
6	−0.241*	0.368**	−0.347**
7	−0.099	0.446**	−0.428**

Abbreviations (TAV_3_, HDAS, BOS_4_, TAV_4_) are the same as in Table 1.

** p<.01.

* p<.05.

+ p<.1.

To sum up, for most of the participants, the specific behavioral change of the upper body (i.e. TAV_4_), rather than inadequate leg movement, was essential for the perception of the odd sensation. We therefore conclude that the perception of the odd sensation is little induced directly by inappropriate lower limb movement, but is dominantly induced by upper body behavioral change.

## Discussion

This study demonstrated that the odd sensation surely emerged in a stopped-escalator situation, but not in the wooden-stairs situation. In addition, we found the non-reduction of the odd sensation in the SE3-B condition (the stopped-escalator situation after two trials on wooden stairs). These results suggest that it is not step-height but stepping on an escalator itself that is essential for the emergence of the odd sensation. Statistical path analysis using a structural equation model further demonstrated the perception of the odd sensation is directly associated with upper body movement (i.e. postural forward sway), which was escalator-specific motor behavior and appeared *after* but not before participants had stepped onto a stopped escalator. This suggests that the prerequisite for the emergence of the odd sensation is an inappropriate motor behavior against the current situation resulting from the habitual motor behavior for a *moving* escalator. People may not be able to suppress this behavior despite completely understanding the current stopped escalator situation.

### Odd sensation emerges according to the visual context, but not the step-height

This study clearly showed that the odd sensation was not an unfamiliar sense as a result of unfamiliar action towards the peculiar step-height. Instead, we found that the postural forward sway after stepping (TAV_4_), which appears to be the consequence of the subconsciously triggered habitual escalator specific motor program, is highly associated with the odd sensation. These results suggest that the odd sensation would not be a phenomenon that simply occurs concurrently with motor actions (i.e., not a sensation due to a simple unfamiliarity with encountering a stopped escalator), but would be a sensation resulting from the discordance between the motor intention for the perceived current external situation (the escalator is stopped) and the actual movements emerging from the subconsciously triggered habitual motor program.

The conflict between the motor intention and the sensory outcome would be the important factor for the emergence of some kind of strange/peculiar feeling. Fink et al. [8] generated such conflict by producing incongruence between the visual feedback of participant's hand and his/her action intention, and showed that participants felt “strangeness/peculiarity” as the extent of the conflict. Although it remains unclear whether this feeling is identical to the odd sensation in this experiment, such conflict would be the necessary condition for the emergence of the odd sensation whether or not sensory outcome is externally manipulated or internally and subconsciously modified (see also last section).

### Consciousness cannot completely dispel the subconscious motor process

Why did participants perform appropriately before stepping and then inappropriately after stepping? The motor behaviors before stepping might be due to the conditions prior to the initiation of movement. In our study, the escalator was already moving prior to task initiation in the moving-escalator situation (ME1–ME 5 conditions in both session), and, of course, it was stopped in the SE conditions. Participants would therefore easily be able to switch their planning according to the experimental situations and could perform properly according to the situation (i.e., moving or stopped) before stepping. Furthermore, the information about whether an escalator is moving or stopped would be the most salient and beneficial for the participant before stepping.

Conversely, a previous motor adaptation task (e.g. [3]) has produced different results. Specifically, after participants had stepped (even just one trial, [4]) onto a moving platform, an inappropriate increase in walking velocity *before* stepping and subsequent postural forward sway were observed when they stepped onto a stopped one. The sled movement in these previous studies [3], [4] was triggered by an optical switch that detected participants' legs, so a view of sled motion was not provided until that trigger. Therefore, the participants in that study could prepare some motor set for the potential sled movement even when an unequivocal warning that sled was not going to move was given. This motor set might have resulted in the increase of walking velocity before they had stepped onto the stopped sled.

The escalator-specific behavioral properties appeared only after the SE1 condition, not after the WS1 condition, although both conditions were identical in terms of the immediate subsequent trial in the ME5 condition. Furthermore, the escalator-specific motor behavior (i.e. TAV_3_, HDAS, TAV_4_ and BOS_4_) in the SE3-B condition showed significant differences from the WS1 and WS2 conditions (Fig. 5). These findings imply that behavioral properties of stepping onto a stopped escalator do not simply reflect a mere aftereffect of motor-specific adaptation [9] or of sensory association adaptation [10], or an aftereffect of “short-term” adaptation specific to a moving escalator as in the mobile sled task in [3]–[5]. Rather these behavioral properties reflect the emergence of a habitual escalator-specific motor program tightly coupled with cues taken at the moment of stepping onto the escalator (e.g. visual, somatosensory, and/or sole's cutaneous information).

The current study clearly demonstrated that the habitual escalator-specific motor program emerged after stepping while one performed properly according to the external situation before stepping. Such motor actions are examples of the emergence of subconscious motor control *inconsistent with* conscious awareness of the current situation, whereas previous studies highlighted that flexibility to switch between the automatic (subconscious) mode and controlled (conscious) one according to the encountered situation, implying even automatic mode was controlled purposively [11]–[13].

### Why does postural forward sway mainly contribute to the odd sensation?

We found kinematic chains after stepping, indicating the higher the heel was raised, the larger the postural forward sway became. Did the postural forward sway passively occur due to the loss of footing? This is unlikely because the tilting angle after stepping increased progressively. No reactive responses [14] for the prevention of postural imbalance were seen, and the tilting angle in the SE1 condition finally became comparable to that in the ME5 condition approximately at the heel strike of the fifth step (see Fig. 3E). Those behavioral properties suggest that an active postural forward sway would occur as a consequence of the habitual manner as described by Graybiel: “habits are sequential … behaviors elicited by external or internal triggers that, once released, can go to completion without constant conscious oversight” ([15], p. 361). In summary, the kinematic chains would reflect the subconscious emergence of the habitual escalator-specific motor program, by which the generated movement does not accord with the external environment (stopped escalator), resulting in the emergence of the odd sensation.

Concerning the odd sensation, why did participants not feel the odd sensation due to the inappropriate motor behavior of lower limbs (all participants except one) but feel it with the postural forward sway? One possible reason is the temporal order of the events. Since the participants reported their sensation right after each trial, the subjective sensation could be strongly affected by the closest behavioral event of the postural forward sway. The other possible reason is a difference in the manner of control: limb movement (e.g., lower limb movement for foot clearance) is controlled by a voluntary component (although an automatic component also exists) with action intention, while posture is controlled mainly in an automatic fashion [16]–[18]. This difference in the manner of control could reflect a difference in the “interpretation” (attribution, see the last part of Discussion) of action error. That is, inappropriate motor behavior of lower limbs could be easily misinterpreted as an action error caused by one's own voluntary motor control. This lower limb movement therefore would not be tightly associated with the odd sensation. On the contrary, participants could not properly interpret why postural forward sway occurred, which cannot be attributed to voluntary action because of automatic postural control. Recently, Bunday and Bronstein [19] showed that even participants devoid of vestibular function still reported a sensation similar to the odd sensation when stepping onto a stopped escalator in their locomotor adaptation task, suggesting that the vestibular system itself does not seem to be essential for perceiving this sensation. We need to further clarify the odd sensation generation process in relation to the automatic and voluntary motor controls in future studies.

### Potential conflict for the odd sensation when prediction is betrayed

Participants consciously understood that the escalator was stopped, so it is plausible that their sensory prediction after they had stepped onto it would be similar to that they had stepped onto the wooden stairs, because the objects to be stepped onto were stationary in both situations. This sensory prediction is mismatched with actual sensory consequence because of the subconscious emergence of the habitual escalator-specific motor program. The mismatch would result in the feeling that we did not properly perform.

This schema seems to be in line with recent motor control theory focusing on an internal model [20]–[23], but can this schema fully explain the emergence of the odd sensation when stepping onto a stopped escalator? In other words, is feeling of improper performance a sole origin for the odd sensation? As briefly discussed above, we speculate that an additional factor is required in order to produce the odd sensation; difficulty in attributing our inappropriate motor behavior to exogenous and endogenous events. As mentioned in Introduction, when we lose our footing (i.e. make a motor error), we are able to attribute this motor error to a self-prediction error caused by our misperception of the external world (exogenous event) or by misgeneration of the motor program (endogenous event).

On the contrary, the inappropriate motor behavior after stepping onto a stopped escalator can hardly to be attributed to either misunderstanding of the situation (exogenous event) or error in the volitional motor program (endogenous event) because our conscious awareness makes us “believe” that our visuomotor system is working properly on the based of our complete knowledge of the situation (i.e., that the escalator is stopped), and this awareness cannot access the emergence of the subconscious motor program. That would be why we feel the odd sensation. Such inability of conscious awareness to access the subconscious motor control would imply the dissociation between declarative and procedural systems as previous studies have suggested [3], [24]–[26].

Cognitive psychology studies have demonstrated how people attribute and evaluate actions in various situations (e.g., [27]), but the situation of attribution difficulty (or loss) and its accompanying odd sensation have not been focused on. The term ‘action attribution’ in motor control studies has been used to refer the origin of an action to its proper agent (i.e., oneself or another person), and the ability of action attribution appears to hinge on the sense of agency, which is the sense of controlling events in the external world [6], [28]. Considering these previous studies, we will further focus on “confidence level (reliability)” of both the internal motor command and external environment as the sources of action attribution. In addition to the growing evidence of the awareness of action [29]–[31], further research of the odd sensation involved in action will contribute to revealing the interaction mechanism between conscious and subconscious processes

## Materials and Methods

### Participants

Seven males (21–44 years of age, mean age = 31.3±7.3 years) participated in the experiment. All participants reported normal or corrected-to-normal vision and none of them had any motor or sensory abnormalities. Participants reported, before the experiment, that they had many opportunities to step onto a moving escalator in their daily life. They also had stepped onto a stopped escalator at least a few times (e.g. at train stations after midnight) and had indeed felt the odd sensation. They gave informed consent to participate in the study, which was approved by the NTT Communication Science Laboratories Research Ethics Committee.

### Apparatus

The escalator used (800EX-EN, Hitachi, Ltd., Tokyo, Japan) is installed inside an NTT building (see Fig. 1A). Escalator velocity in the depth direction is 0.5 m/s; that in the height direction (except initial elevation) is 0.25 m/s. To investigate the effect of the stopped escalator's step-height itself on behavioral properties and odd sensation, custom-made wooden stairs with an approach that duplicates the physical dimensions of a stopped escalator (i.e. shorter first step) were prepared (the wooden stairs have four steps and no hand rail). The wooden stairs were set near the escalator.

Reflective 30 mm markers were attached at four locations on the body as follows; C7 of spine, basis ossis sacri (BOS), right and left heels (Tuber calcanei) (See Fig. 1B). These markers were recorded with a 3D motion capture system (ProReflex, Qualisys, Sweden) at a frequency of 250 Hz. Three infrared cameras were used to record of movements towards the escalator and wooden stairs.

### Procedure

Participants started their self-paced walking to the escalator or wooden stairs after an experimenter's cue. They were not instructed to initiate their gait with a certain leg. Six participants started their gaits with the right leg, and one participant started with the left leg. There were three situations in the experiment: walking toward and stepping onto a moving escalator (ME), a stopped escalator (SE), and wooden stairs (WS). The task was walk to forward for six steps and stop, aligning both legs at the last step (Fig. 1A). Participants were asked to score the extent to which they felt some kind of odd sensation after each trial for the stopped escalator and wooden stairs on a five point-scale (5 indicating very strong and 1 (almost) none).

The experiment consisted of two sessions (sessions *A* and *B*), each divided into two subsessions (A1, A2, B1, B2). In a block of session *A*, five consecutive ME trial (ME1–ME5) were followed by three consecutive SE trials (SE1–SE3). In a block of session *B*, participants performed five ME trials, two WS trials (WS1, WS2), and finally a SE trial (SE3-B) sequentially. Our primary interest was the behavioral properties and the score of the odd sensation for the stopped escalator. However, participants' performance and the perception of the odd sensation would adapt soon due to the repetition of the stopped-escalator experience, so we aimed to maintain specific motor behaviors and the odd sensation in the stopped escalator by inserting the ME trials in both sessions. In other words, the ME trials are not adaptation tasks in the sense of the typical adaptation experiment paradigm, because stepping onto a moving escalator is already a highly habituated action. Each subsession (i.e. A1, A2, B1, B2) consisted of eight blocks. Three participants did the experiment in the order A1, B1, B2, A2, and the rest of them did it in the order B1, A1, A2, B2. Accordingly, a total of 16 blocks were done for each session. For data analysis, each trial was grouped by the trial order in each session, and the grouped trials (i.e., 16 trials) in each condition determined by the session and trial order were named as follows: SE1, SE2, SE3 conditions in session *A*, WS1, WS2, SE3-B conditions in session *B* (See Fig. 1C). ME5 condition in session *A* was also analyzed as the typical condition of the habitual action of stepping onto a moving escalator.

### Data processing and analysis

The odd sensation scores reported on the 5-point scale (see Procedure subsection above) was regarded as the interval scale [32]. Our interests are (i) whether the odd sensation certainly emerged in a stopped escalator situation, and if so, (ii) whether the perception of the odd sensation varied according to the repetition of trials (i.e., the difference among the scores in the SE1, SE2, and SE3 conditions), and (iii) whether the odd sensation varied according to given contexts (i.e., the difference between those in SE1 and WS1, in SE2 and WS2, and in SE3 and SE3-B). The mean data were analyzed with a two-way repeated measures ANOVA with session (sessions *A* and *B*) and trial order (first, second, and third trials after ME trials). If we found an interaction, the simple main effect was examined. Tukey's HSD procedure was used for post-hoc comparison of means (alpha level = .05).

For motion data analysis, each marker data point was filtered offline using a fourth-order Butterworth filter (double sided) with a cutoff frequency of 10 Hz and then differentiated to obtain velocity and acceleration. BOS velocity in the horizontal direction (BOS velocity) was calculated as walking velocity. As indices of postural sway, we used tilting angle (TA) defined as the angle made by the line from C7 connecting the BOS and gravitational line and tilting angle velocity (TAV) obtained by differentiating the TA (Fig. 1B). There were two steps in the analysis. First, we aimed to identify the basic behavioral properties of the stopped escalator by comparing the mean data (see below) among ME5 (in session *A*), SE1, WS1 conditions. The mean values were analyzed with an ANOVA with the condition (ME5, SE1, WS1) as within-participant factors. Next, if the values showed the significant differences between SE1 and WS1 conditions, mean data were analyzed with a two-way ANOVA with session (sessions *A* and *B*) and trial order (first, second, third trials after trials of moving escalator) as within-participant factors. If we found an interaction, the simple main effect was examined. Tukey's HSD procedure was used for post-hoc comparison of means (alpha level = .05).

To prevent increases in data variability by the deviation of the action phase, the BOS velocity, TA, and TAV were aligned by the times of each heel strike of the second, third, and fourth steps, and we analyzed the aligned data until the phase around the heel strike of the next step, as shown in Fig. 3. The alignment time was defined by the zero-crossing of heel vertical velocity (from negative to positive) of each step. We defined the alignment time of each step as 0 s, and calculated mean values of a 0.2 s time window on the basis of the alignment of each step. Window T represents time window T ±0.1 s. As for BOS velocity, we set windows from −0.3 to 0.6, at every 0.1 s, on the basis of the alignment of the second step as depicted in Fig. 3A, and windows from 0 to 0.5 on the basis of the heel strike of the third and fourth steps respectively, as depicted in Figs. 3B and 3C. Note that windows 0.6 in Fig. 3A and window 0 in Fig. 3B, and window 0.5 in Fig. 3B and window 0 in Fig. 3C were temporally overlapped. From window 0.2 based on the alignment of third step, spontaneous BOS velocity in the ME5 condition was calculated by subtracting escalator speed itself (i.e. 0.5 m/s) from the measured BOS velocity, and we used this velocity for the analysis. Mean TAs and TAVs of 0.2 s time windows were calculated (i.e., windows 0–0.5, from the alignment of the third and fourth steps).

We also analyzed leg movements to the heel strike on Step 1 (Fig. 1A) to investigate whether any specific motor behavior influenced by the step-height appeared or not. For this purpose, heel's downward approach to strike (HDAS) was computed using heel vertical velocity as follows. First, the data were aligned by the maximal heel vertical velocity of the fourth step (in this case, time 0 s indicated this alignment time). Second, in the phase of [0.3–0.5] with this alignment, we detected negative values of the velocity from 0.3 s to the zero-crossing point (i.e., the heel strike of the fourth step) and computed the difference between the negative value and zero at each sampling point. Finally, the summation of these differences was divided by the sampling frequency (i.e. 250) in each trial, and we averaged these values in each condition as the index. We did not calculate HDAS in the ME5 condition, because the situation of the heel strike was different from those in other conditions due to the elevation of the moving step. We therefore just compared the values between the SE1 and WS1 conditions.

Finally, we investigated whether the perception of the odd sensation is associated with any behavioral properties in the stopped escalator, and whether there is a kinematic chain between lower limb and upper body movements. For this purpose, we introduced path analysis using structural equation modeling (SEM) [7].

Path analysis is a statistical approach for exploring causal relationships among measured variables. On the basis of the theory or researchers' hypothesis, a model is depicted by path diagrams, which are designed to show variables interconnected with lines to indicate causal flow (see center panel of Fig. 5). The model is evaluated using SEM goodness-of-fit tests to determine if the pattern of variances and covariances in the data was consistent with the model specified. We used the Goodness of Fit Index (GFI), Comparative Fit Index (CFI), Root Mean Square Error of Approximation (RMSEA) as the indices of goodness-of-fit. Values of GFI and CFI greater than 0.9 and a value of RMSA about .05 or less were considered to be well fitting.

The hypothesized path model is shown in the center panel of Fig. 5. We have the assumption that the behavioral properties showing significant differences between SE1 and WS1 conditions would contribute to the emergence of the odd sensation. From such data, we selected four behavioral events, taking into account the temporal phases in which the events occurred: TAV at 0.1 in Fig. 3D (i.e., TAV immediately after stepping; TAV_3_ in Fig. 5), HDAS (i.e., leg movement before the heel strike of the fourth step), BOS at 0.1 in Fig. 3C, and TAV at 0.1 in Fig. 3E (i.e. motor behaviors immediately after the heel strike of the fourth step; BOS_4_ and TAV_4_ in Fig. 5). We adopted the TAV as the index of postural movement because the TAV is more suitable for detecting the transient behavioral changes than TA. Furthermore, the tilting angle around at the heel strike of the fifth step was assumed to be the subsequent event to BOS_4_ and TAV_4_, so we did not adopt this movement as the variable for model simplicity. The odd sensation score was regarded as an interval scale again [33]. The data in all the SE conditions (SE1, SE2, SE3, and SE3-B) were analyzed for the path analysis, and we focused on the standardized path coefficients between variables. Standardized path coefficients indicate the relative effect of variables within the model.
